# Reproducibility of Diagnostic Criteria for Ventricular Neurocysticercosis

**DOI:** 10.4269/ajtmh.17-0724a

**Published:** 2017-12-06

**Authors:** Agnes Fleury, Arturo Carpio, Matthew L. Romo, Daniel San-Juan, Josemir W. Sander

**Affiliations:** Instituto de Investigaciones Biomédicas Universidad Nacional Autónoma, México Distrito Federal, México. Instituto Nacional de Neurología y Neurocirugía Secretaría de Salud, México Distrito Federal, México E-mail: afleury@biomedicas.unam.mx; Facultad de Ciencias Médicas Universidad de Cuenca Cuenca, Ecuador G.H. Sergievsky Center Columbia University New York, New York E-mail: arturocarpio@etapanet.net; Facultad de Ciencias Médicas, Universidad de Cuenca, Cuenca, Ecuador CUNY Graduate School of Public Health and Health Policy, New York, New York E-mail: Matthew.L.Romo@gmail.com; Instituto Nacional de Neurología y Neurocirugía, Secretaría de Salud, México, Distrito Federal, México E-mail: pegaso31@yahoo.com; NIHR University College London Hospitals Biomedical Research Centre, UCL Institute of Neurology, London, United Kingdom Stichting Epilepsie Instellingen Nederland (SEIN), Heemstede, The Netherlands E-mail: l.sander@ucl.ac.uk

Dear Sir,

We read with interest the manuscript by Bustos et al.,^1^ which provided data on the validity of their own diagnostic criteria for ventricular neurocysticercosis (NCC).^2^ We agree that improving the diagnosis and management of people with NCC is a priority, and any tools that would help to improve this are always welcomed.

In their discussion, the authors state that when our previously published validated diagnostic criteria^3^ were applied to the same cases, they found poor specificity (39%). We were surprised by this, as this step was not mentioned in the Methods section—nor were any results provided. We can, therefore, surmise that this seems to have been done ad hoc. We were also bewildered by the specificity value provided and attempted to reproduce specificity for both criteria (as data were provided).

To estimate specificity, the cases used by Bustos et al.^1^ were independently reviewed by two of our team (A. F., D. S.-J.), and they obtained different results than those reported (Table 1). Of the 40 cases that we retrieved, the specificity for each of the evaluations of our criteria was 82.5% and 78.1% (confirmed by an external statistician). We were also surprised that we could not reproduce the specificity reported by the authors for their own criteria as we again found differing values (see Table 1). We did not attempt to estimate sensitivity as it seems only reasonable to assume that they are also not reproducible.

**Table 1:** Specificities of Carpio et al. and Del Brutto et al. criteria independently evaluated

	Specificity (95% CI)	
	Evaluator 1	Evaluator 2
Carpio et al.^3^ criteria	78.1% (62.4–89.4%)	82.5% (67.2–92.7%)
Del Brutto et al.^2^ criteria	31.7% (18.1–48.1%)	55.0% (38.5–70.7%)

CI = confidence interval.

Our finding generated concerns as to why the results of Bustos et al.^1^ are not reproducible. One possibility is that their review of cases and controls was not blind as this is not mentioned in their methods. Of course, the authors may well have an alternative explanation for this lack of reproducibility, and we look forward to receiving such an explanation if this is the case.
